# Self-motion perception without sensory motion

**DOI:** 10.1007/s00221-022-06442-3

**Published:** 2022-08-20

**Authors:** A. J. C. Reuten, J. B. J. Smeets, M. H. Martens, J. E. Bos

**Affiliations:** 1 Department of Human Movement Sciences, Vrije Universiteit Amsterdam, Amsterdam, The Netherlands; 2 Human Performance, The Netherlands Organization for Applied Scientific Research (TNO), Soesterberg, The Netherlands; 3 Traffic and Transport, The Netherlands Organization for Applied Scientific Research (TNO), The Hague, The Netherlands; 4 Department of Industrial Design, Eindhoven University of Technology, Eindhoven, The Netherlands

**Keywords:** Self-motion perception, Vestibular cognition, Neural store, Psychogenic dizziness, Mal de débarquement

## Abstract

Various studies have demonstrated a role for cognition on self-motion perception. Those studies all concerned modulations of the perception of a physical or visual motion stimulus. In our study, however, we investigated whether cognitive cues could elicit a percept of oscillatory self-motion in the absence of sensory motion. If so, we could use this percept to investigate if the resulting mismatch between estimated self-motion and a lack of corresponding sensory signals is motion sickening. To that end, we seated blindfolded participants on a swing that remained motionless during two conditions, apart from a deliberate perturbation at the start of each condition. The conditions only differed regarding instructions, a secondary task and a demonstration, which suggested either a quick halt (“Distraction”) or continuing oscillations of the swing (“Focus”). Participants reported that the swing oscillated with larger peak-to-peak displacements and for a longer period of time in the Focus condition. That increase was not reflected in the reported motion sickness scores, which did not differ between the two conditions. As the reported motion was rather small, the lack of an effect on the motion sickness response can be explained by assuming a subthreshold neural conflict. Our results support the existence of internal models relevant to sensorimotor processing and the potential of cognitive (behavioral) therapies to alleviate undesirable perceptual issues to some extent. We conclude that oscillatory self-motion can be perceived in the absence of related sensory stimulation, which advocates for the acknowledgement of cognitive cues in studies on self-motion perception.

**Supplementary Information:**

The online version contains supplementary material available at 10.1007/s00221-022-06442-3.

## Introduction

Vestibular and visual signals inform us about our self-motion, for example when moving back and forth on a swing. Our perception of such self-motion is not only based on this sensory input, as cognition has been demonstrated to play a role as well (Ferrè and Haggard 2020; Ferrè and Harris 2015; Mast and Ellis 2015). Specifically, several studies demonstrated that mental imagery (Mertz et al. 2000; Nigmatullina et al. 2015), a priori motion expectations (Ellis et al. 2017), and contextual information (D’Amour et al. 2021; Riecke 2009; Wertheim et al. 2001) modulated self-motion perception of a physical or visual motion stimulus. These studies all concerned experiments with motion stimuli and thus reflect modulations of a percept of self-motion that is elicited by sensory stimulation. Because we are not aware of any study on self-motion perception without a motion stimulus, we performed a study investigating whether cognitive cues can elicit a percept of self-motion in the absence of sensory motion. In specific, we minimized physical (inertial), visual, somatosensory and auditory cues about self-motion.

**Fig. 1:**
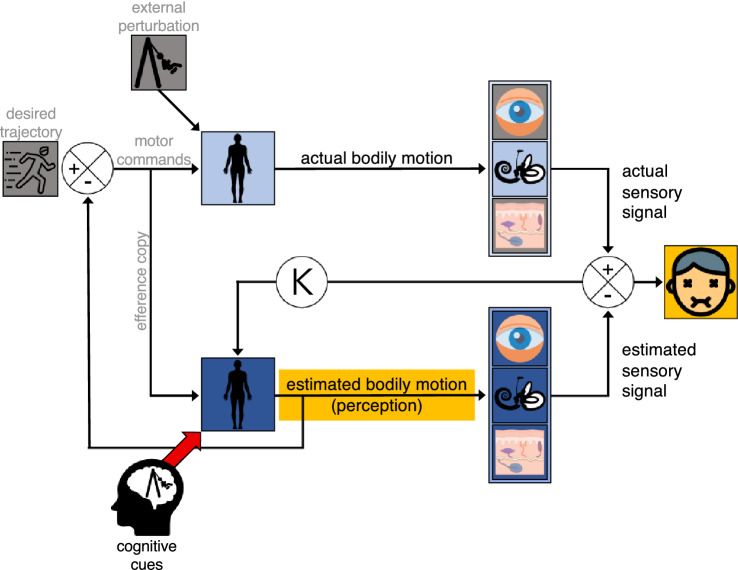
Simplified model of sensorimotor control, including the perception of motion and the origin of motion sickness (based on Bos et al. 2008; Kuiper 2019; Oman 1991; Reason 1978; Wolpert et al. 1998). Light blue boxes represent the actual motor and sensory systems; the dark blue boxes represent the internal models of these systems. In our study, we minimized physical (active and passive), visual, somatosensory and auditory cues about self-motion (in grey). The remaining inputs are cognitive and vestibular cues on (the lack of) motion. Our measures of interest are perceived self-motion and motion sickness (yellow elements)

When modeling sensorimotor control, authors frequently include internal forward models that process an efference copy of motor commands (dark blue boxes in Fig. 1; Oman 1982; Popa and Ebner 2019; Wolpert et al. 1998). The internal model of the bodily dynamics (dark blue box) estimates the bodily motion that would result from the motor commands. This estimation controls our perception of self-motion. Under optimal conditions, it equals the actual self-motion produced by the real body (light blue box). As this prediction lacks the delay and other peculiarities of the sensorimotor system, it is the best input for feedback control of self-motion. To ensure that this estimate is indeed accurate, this signal is fed to the internal model predicting the sensory signals (visual, vestibular, and somatosensory; dark blue). If there is a difference between the resulting integrated estimated and actual sensory signal, their discrepancy will be used to update the internal model and hence the estimated bodily motion. The discrepancy itself is assumed to cause motion sickness (Reason 1978). The updating of the internal model is weighted relative to the noise of the actual sensory systems by a gain K (Oman 1982; Tanaka et al. 2020, see the ‘Kalman’ gain K in Fig. 1). A high uncertainty about those signals is then accounted for by a low gain and, vice versa, a low uncertainty by a high gain. In the current study, we are interested whether not only efference copies but also motion expectations generated by cognitive cues influence the estimated bodily motion. Given that the updating of the internal model is based on a Kalman gain, motion will only be reported when there is a low signal to noise ratio of the senses. If so, cognitive cues could result in the perception of self-motion in the actual absence of motion.^1^ If this perceived motion is large enough, its difference from the absent sensory signal could accordingly cause individuals to feel motion sick.

Our study thus aims to answer the question whether we can indeed induce a systematic percept of self-motion without sensory motion using cognitive cues. If the answer is affirmative, we can answer a second question: does the neural mismatch between this estimated percept of self-motion and a lack of corresponding sensory signals provoke motion sickness? To that end, we seated blindfolded participants on a swing that remained motionless during two conditions, and additionally provided a noise cancelling headphone and airflow to minimize further sensory cues on motion. The only difference between the conditions was a cognitive induced manipulation of expectations regarding the swing’s motion. In both conditions, we repeatedly asked participants about their perceived oscillatory self-motion and level of motion sickness (see yellow elements in Fig. 1).

## Methods

We exposed blindfolded participants to two conditions on a parallel swing, between which we differently manipulated expectations regarding the motion of this swing (within-participant design). In reality, we only let the swing move with a transient oscillation at the start of each condition. However, in a “**Focus** on motion” condition, we aimed at letting participants believe that the swing was moving for the entire condition. We therefore told participants before the start of this condition that the swing would be oscillating with varying peak-to-peak displacements, and asked them about this motion at regular intervals during the condition. We moreover demonstrated to participants that the swing could move back and forth. In a “**Distraction** from motion” condition, we aimed at letting participants believe that the swing was only oscillating at the beginning of the condition. We therefore told participants before the start of this condition that the swing would come to a stop after an initial perturbation, and distracted them from the swing’s possible motion by asking motion irrelevant questions about pitch differences of a tune during the condition. In summary, the cognitive (non-sensory) cues consisted of (1) instructions about the swing’s motion, (2) a discrimination task with different attentional allocation performed during the conditions, and (3) a demonstration of swing motion.

### Participants

We recruited 24 participants (16 females) from the Vrije Universiteit Amsterdam in The Netherlands, where the experiment was performed. Participants were allowed to participate if they were 18 years or older, had experienced motion sickness in the last 5 years, were free of (self-known) vestibular and auditory complaints, were not pregnant, did not suffer from claustrophobia, and never participated in an experiment on our setup before. Our participants were aged between 18 and 24 years. We have obtained ethical approval from the faculty’s review board (reference number: VCWE-2020-180R1).

### Experimental setup

**Fig. 2:**
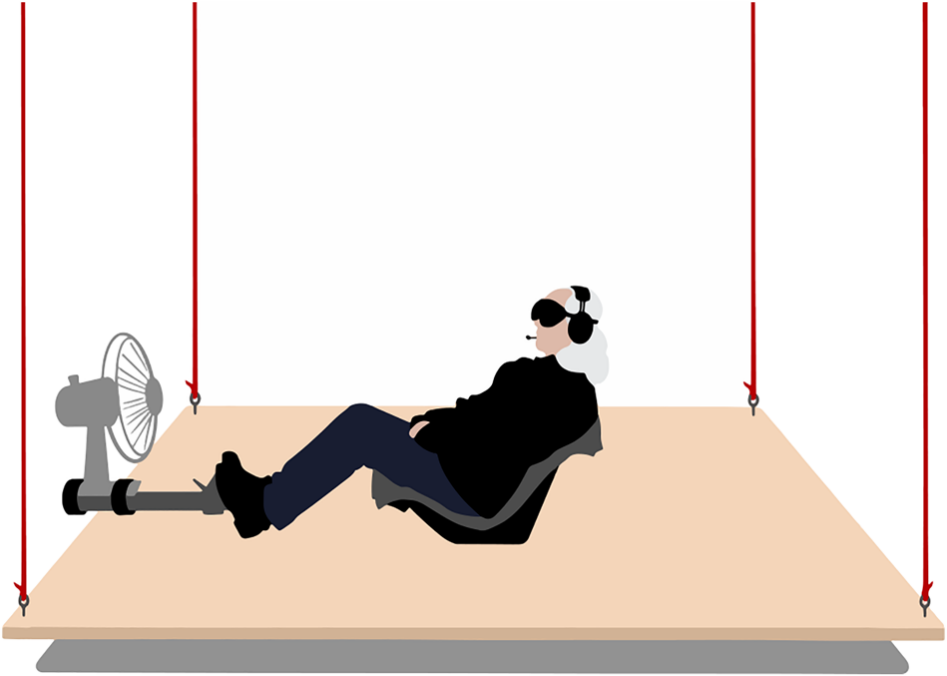
Experimental setup. A participant is seated on the swing, wearing blinding goggles and a noise cancelling headphone to remove external motion cues. We used a swiveling fan to mask airflow and a footrest to support a stable seating position

In both conditions, participants were seated on a parallel swing (Oosterveld 1970). The swing consisted of a 250 × 245 cm platform attached to the ceiling with four 6.65 m ropes (Fig. 2). Given this length, the swing oscillated with a natural frequency of 0.19 Hz when perturbed, close to the peak frequency of motion sickness incidence (Golding et al. 2001; ISO 1997).

**Fig. 3:**
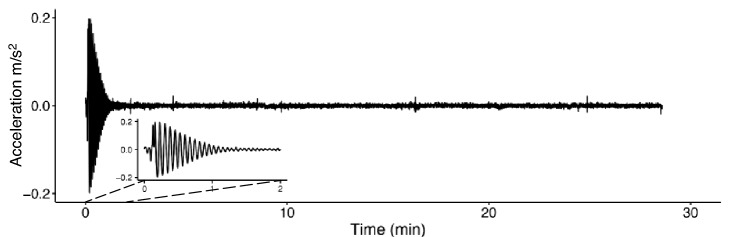
The swing’s acceleration during a typical condition. The swing was released from an initial forward displacement at the start of the condition, resulting in a transient oscillation reaching standstill within 2 min (see inset). The isolated spikes later in the acceleration trace correspond to small body movements of the participant

To support the perception of oscillatory motion in the Focus condition, the experimenter unleashed the swing from a 10 cm forward displacement at the beginning of both conditions. This resulted in a transient oscillation returning the swing to a standstill within 1–2 min (see Fig. 3). To check the swing’s motion, we recorded its acceleration in the longitudinal direction using the accelerometer of a mobile phone, measuring at 20 Hz using MATLAB Mobile for iOS (version 8.4). We detrended the signal and removed the measurement noise using a bidirectional first order low-pass Butterworth filter with a cutoff frequency of 2 Hz. The resulting root mean square acceleration excluding the first 2 min was 0.003 ± 0.001 m/s^2^ (mean ± SD) on average in both conditions. This average is considered well below the threshold for motion perception, assumed between 0.1 and 0.01 m/s^2^ (Griffin 1990). Any percept of oscillatory self-motion can thus not be explained for by physical motion stimulation. We additionally minimized visual motion cues by blindfolding participants for the entire duration of the condition; somatosensory motion cues by airflow generated by a swiveling fan rotating at a frequency of 0.05 Hz, thus uncorrelated to the natural frequency of the swing; and auditory motion cues by a noise cancelling headphone (see also Fig. 2).

### Tasks and measurements

**Fig. 4:**
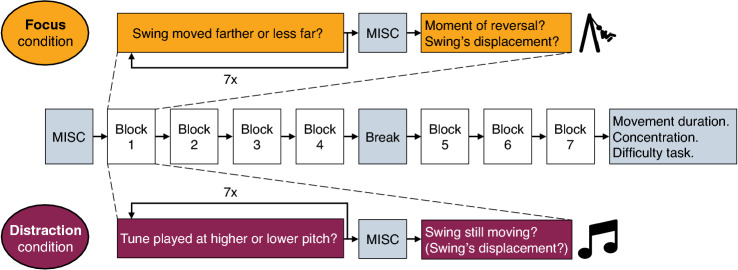
Overview of the rating tasks used in the two conditions. Each block had a duration of 3 to 4 min, the break was 2 min, and the sequence of final questions took 3 min

Both conditions contained seven blocks, a break, and a set of three exploratory questions (see Fig. 4). Each block consisted of a discrimination task that was repeated seven times, followed by a sickness rating using the Motion Illness Symptoms Classification (MISC, Table 1; Bos et al. 2005; Reuten et al. 2021) and two additional questions on the perceived swing motion.

**Table 1:** The Motion Illness Symptoms Classification (MISC) used to assess motion sickness symptomatology (Bos et al. 2005; Reuten et al. 2021)

Class description	MISC
No problems	0
Some discomfort, but no specific symptoms	1
Dizziness, cold/warm, yawning, headache, tiredness, sweating, stomach / throat awareness, burping, blurred vision, salivation, … but no nausea	
Vague	2
Little	3
Rather	4
Severe	5
Nausea	
Little	6
Rather	7
Severe	8
Retching	9
Vomiting	10

In the Focus condition, the discrimination task consisted of seven repetitions of 15 s focusing on the swing’s motion, each followed by the question whether the swing had moved farther or less far as compared to the previous time asked. After participants completed this task and rated their sickness, we asked them to indicate when the swing reversed direction. This question was added to strengthen the participants’ cognitive involvement with the swing’s oscillations. After this, we asked them to indicate the peak-to-peak displacement of the swing’s motion about that moment (further referred to as ‘displacement’). Four participants expressed their doubts on whether the swing was indeed moving. In these cases, the experimenter once used the encouragement “the swing is moving, but the movements may be very small, thus try to pay close attention to them”.

In the Distraction condition, the discrimination task consisted of seven repetitions of 15 s listening to a music clip (Jerry Martin’s “Under Construction”), each followed by the question whether the sample was played higher or lower in pitch as compared to the previous time asked. Pitch height was truly increased or decreased by 4.8 or 9.6% relative to the previous sample (adapted using Audacity 2.4.2.0) aiming to achieve a comparable level of mental workload and task difficulty compared to the task in the Focus condition. After completion of the discrimination task and sickness rating, we asked participants to indicate whether they thought the swing was still moving, and if they did, the second question then was which (peak-to-peak) displacement the swing had about that moment.

The blocks succeeded each other without additional manipulation. To offer participants a break from intensely concentrating, we asked them to perform an alternative task between block four and five. They had to list as many words as possible starting with a certain letter of the alphabet within 1 min. After participants had completed the seven blocks, we asked them three additional questions whilst still being seated on the swing. The first question was which percentage of time they thought the swing had moved (0% = never moved to 100% = always moved). The second question was on their ability to concentrate on the discrimination task (0 = poor to 10 = good). The last question was on the difficulty of the discrimination task (0 = very easy to 10 = very difficult).

### Procedure

After arrival, we instructed participants on the experimental procedure and asked them to sign an informed consent form. Participants filled out the Motion Sickness Susceptibility Questionnaire (MSSQ-Short; Golding 2006) from which we observed that the sample’s susceptibility to motion sickness fell within the 60th percentile. Following completion of the MSSQ-Short, participants performed the two conditions. Because of individual differences in response time and the additional question about the moment the swing reversed direction in the Focus condition, the conditions lasted between 25 and 35 min. We presented the conditions in counterbalanced order with a 45-min break in between, to allow for recovery of motion sickness. To minimize an observer–expectancy effect (see Rosenthal 1963; Rosenthal and Fode 1963), we provided all instructions via pre-recorded audio files, both before and during the conditions. Although we were interested in the effect of motion expectations on self-motion perception and motion sickness, we told participants that we were interested whether their ability to discriminate small differences in displacement and pitch were related. We introduced the MISC as a measure to monitor their level of well-being as it could influence their task performance.

We stopped a condition when a participant rated a MISC class of ≥ 6, which occurred once in the Focus condition and three times in the Distraction condition. After completing the experiment, participants were thanked for their participation and received study credits.

### Data analysis

Our primary dependent variables were the displacement and MISC class participants rated at the end of each block in both conditions. Missing data as the result of the exerted MISC stop-criterium were substituted with the last rated displacement and MISC class. To answer our two questions, we averaged the seven displacements and MISC classes given by each participant in the Focus and Distraction condition and analyzed the within-participant differences using Wilcoxon signed-rank tests (with *α* = 0.05). To explore the data further, we report the averaged within-participant difference between the conditions for various measures, together with the between-participant standard deviation (mean difference ± SD, Focus minus Distraction).

## Results

**Fig. 5:**
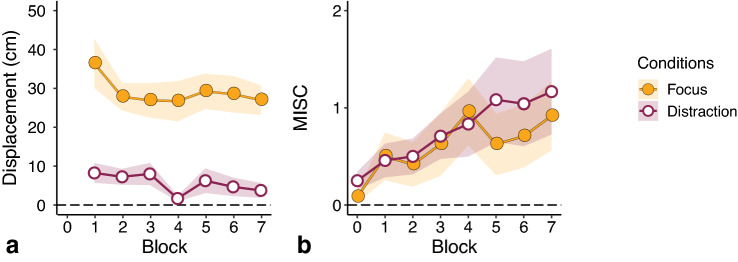
Temporal development of displacement (**a**) and MISC class (**b**) in the Focus and Distraction condition. Each symbol represents the average across participants, with shaded areas representing the standard errors of the mean

We first investigated the development of displacement and MISC class during the conditions (Fig. 5). There was a clear and consistent difference in the reported displacements between the Focus and Distraction condition (Fig. 5a), implying that our manipulation on motion expectations was effective. Regarding the MISC classes, we did observe an increase in motion sickness as the conditions continued. However, this increase in sickness was very limited: the average maximum MISC class corresponded to some discomfort without symptoms. Most importantly, there was no difference in sickness level between the conditions (Fig. 5b). We present the temporal response traces per participant in Supplementary Fig. S1.

**Fig. 6:**
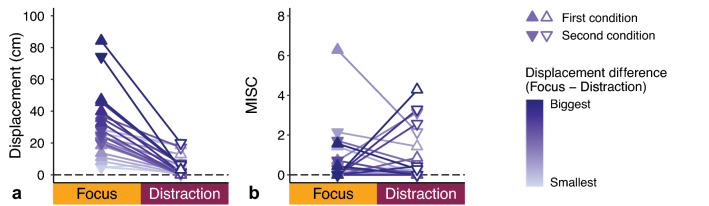
Displacements (**a**) and MISC classes (**b**) reported in the two conditions. Each symbol represents the average value of an individual participant in that condition. We used a gradient to contrast the participant with the biggest displacement difference between conditions (in dark purple) to the participant with the smallest displacement difference (in light purple). To visualize a possible effect of condition order, we used different symbols indicating the order of conditions for each participant

Because we were mainly interested in a comparison within participants, we averaged the displacements and MISC classes between conditions and plotted the resulting values per participant in Fig. 6. We observed a systematic difference in the percepts between the conditions: all participants (except one) reported a larger average displacement in the Focus compared to Distraction condition (Fig. 6a). On average, the difference was 23.6 ± 17.7 cm, which was significant (*W* = 1, *p* < 0.001, *r* = − 0.87). In contrast, the MISC classes were very similar between the two conditions (average difference − 0.1 ± 1.7; *W* = 92.5, *p* = 0.936; Fig. 6b), without any apparent effect of condition order. We explored whether there was a correlation between displacement and MISC class independent of condition, but observed no such evidence (see Supplementary Fig. S2a).

**Fig. 7:**
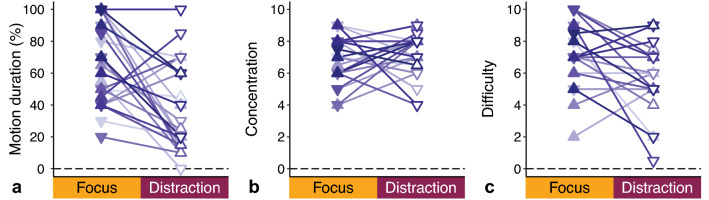
Responses to the questions asked at the end of each condition. **a** Reported motion duration of the swing (0% = never moved to 100% = always moved). **b** Reported ability to concentrate (0 = poor to 10 = good) on the discrimination tasks performed during the conditions. **c** Reported difficulty (0 = very easy to 10 = very difficult) of these discrimination tasks. Details as in Fig. 6

At the end of each condition, we asked participants to indicate which percentage of time they thought the swing had moved (Fig. 7a). The majority of participants indicated that the swing moved longer in the Focus compared to Distraction condition, with a mean difference of 27 ± 35%. Evidently, many reacted with surprise upon hearing that the swing had only moved at the beginning of both conditions. We also explored a correlation between motion duration and MISC class, but again observed no evidence (see Supplementary Fig. S2b). We additionally asked participants to report their ability to concentrate on the discrimination task and to indicate how difficult they thought this task was. Most of them indicated that they were well able to concentrate on both tasks (mean difference − 0.2 ± 2.0; Fig. 7b). The responses for task difficulty were more variable across participants, but similar in the two conditions (mean difference 1.4 ± 2.7; Fig. 7c). On average, 65 ± 18% of the given answers for the pitch discrimination task in the Distraction condition were correct. For the motion discrimination task in the Focus condition, participants also responded close to chance: they reported that the swing was moving with a larger displacement in 44% of the time, and with a smaller displacement in 56% of the time. These numbers indicate that we succeeded in designing tasks that were comparable in difficulty.

## Discussion

In this study, we first investigated whether cognitive cues manipulating motion expectations could elicit a percept of oscillatory self-motion in the absence of sensory motion. If so, we could use this percept to investigate if the resulting mismatch between estimated self-motion and a lack of corresponding sensory signals is motion sickening. To that end, we seated blindfolded participants on a swing that remained motionless during two conditions, apart from a deliberate perturbation at the start of each condition. The two conditions only differed regarding cognitive cues suggesting either a quick halt (“Distraction”) or continuing oscillations of the swing (“Focus”). This manipulation let participants perceive that the swing oscillated with larger peak-to-peak displacements and for a longer period of time in the Focus condition. As the size of the perceived displacement was rather limited, the reported levels of motion sickness were low, with no observable difference between the two conditions.

Our interpretation of the experimental results is that participants can perceive oscillatory self-motion in the absence of sensory stimulation related to motion. Of course, the participants sensed a transient oscillation for the first 1–2 min in both conditions. As this motion had stopped well before the end of the first block, all reports on the perception of motion were made without sensory motion. Though participants shifting position caused some acceleration (see Fig. 3), the reported displacements were consistent across the whole condition and should thus be considered independent of these distortions. A limitation of this study is that the perceived motion was of a displacement too small to elicit motion sickness. The average reported displacement in the Focus condition was 29 cm; estimated to result in a sickness incidence of only 1% when assuming a physical motion stimulus of 30 min (ISO 1997).^2^ This prevents us from answering our second question of interest. It might be worthwhile to explore whether our paradigm could yield the perception of larger displacements by changing some aspects of the experiment.

One aspect that may have limited the reported displacements is the positioning of the experimenter’s desk one meter in front of the swing. Participants might have assumed in their responses that the swing would remain at a safe distance from the desk. After all, Wertheim et al. (2001) demonstrated that a priori knowledge on motion direction had likely guided participants’ responses in other studies. Follow-up studies should be aware of this possible consequence and may expose participants to the experimental setup only when blindfolded.

One might be concerned that the reports of swing motion reflect our instructions, instead of reflecting a true belief that the swing was moving. Some parts of the communication with participants contradict this claim. For instance, four participants openly expressed their doubts about whether the swing was really moving. We probed them to pay close attention to the possibly very small oscillations, after which three participants reported a 1- or 2-cm displacement, and the fourth 10 cm. These reported displacements reflecting the instructions were much smaller than the average displacement of 29 cm reported in the Focus condition. Moreover, when also considering the surprised reaction of other participants upon receiving the debriefing information, we deem it unlikely for an observer–expectancy effect to explain the observed difference in reported displacement between the Focus and Distraction condition.

It may seem surprising that participants perceived some oscillatory self-motion in the Distraction condition as well. There are some aspects in the design of our experiment that might have caused this percept. First of all, we instructed participants that the swing would oscillate at the beginning of the condition, and asked them when they thought the swing stopped moving. Secondly, participants experienced that the swing could oscillate as the platform moved when getting seated. Thirdly, the frequency of respiration in rest may come close to the natural frequency of the swing, which might generate a sense of motion in a state of introspection. Finally, sensory signals are noisy, and could incorrectly register some sense of self-motion.

The reported level of motion sickness developed equally in both conditions until block four (Fig. 5b), after which the average MISC class steadily increased in the Distraction condition whilst it temporarily decreased in the Focus condition. After this brief reduction, the motion sickness scores regained their initial increase. This temporary drop might be related to the break provided between blocks four and five, although it is unclear why it is then only affecting motion sickness in the Focus condition.

Despite all participants (except one) reporting larger displacements in the Focus as compared to Distraction condition, there were rather large between-participant differences in the size of the reported displacements (Fig. 6a). In fact, mean differences were ranging from − 2 to + 71 cm (Focus minus Distraction). We wanted to demonstrate that our analysis was not driven by a few extreme responses, yet we observed that none of the percepts met the common outlier criterion of three times the standard deviation. The large differences might reflect underlying trait variations in phenomenological control, which is the ability to construct an experience that meets certain expectancies (Dienes et al. 2020).

An analogy to the observed percepts of self-motion may be given by tinnitus, the perception of sound in the absence of an acoustic stimulus. Apart from a sensory defect, its occurrence can also be explained by neural structures generating the sound. This latter explanation has gained recognition and already resulted in the development of cognitive behavioral therapies (Langguth et al. 2013). Our results may point in the same direction when considering diseases like mal de débarquement syndrome (Mucci et al. 2018) or persistent postural–perceptual dizziness (Dieterich and Staab 2017), where patients report persistent motion sensations or dizziness in the absence of related sensory input.

Although our participants experienced similar levels of motion sickness in the two conditions, the reported percepts of oscillatory self-motion show some support for the existence of internal models. They may explain the effectiveness of anticipatory cues that communicate upcoming vehicle motion in reducing motion sickness (e.g., Diels and Bos 2021; Feenstra et al. 2011; Hainich et al. 2021; Kuiper et al. 2020). Such cues allow for a more accurate prediction of self-motion, thereby minimizing a (potential) neural conflict and hence the development of motion sickness.

Different from previous studies which showed that cognition can modulate the perception of self-motion elicited by sensory stimulation, we here demonstrated that cognitive cues can induce percepts of oscillatory self-motion in the absence of sensory motion. We argue that the strong influence of cognitive cues on self-motion perception may be explained by internal models of the motor and sensory systems within our central nervous system that provide predictions of self-motion and sensory signals. This finding supports the assumption that undesirable perceptual issues can be somewhat alleviated by cognitive (behavioral) therapy. In any case, our results show that studies on self-motion perception require a detailed description of experimental details such as task instruction, attentional allocation and distraction, and demonstration of motion stimuli that might involve cognitive cues.

## Supplementary Information

Below is the link to the electronic supplementary material.

Supplementary file1 (PDF 198 KB)

## Data Availability

All data and code are publicly available via the Open Science Framework (https://osf.io/q7wux/).

## Footnotes

1 Such an ‘illusory’ percept of self-motion might remind some readers of vection. For the reason that terminology on self-motion perception—including the definition of vection—is ambiguous (Palmisano et al. 2015; Soave et al. 2021), we decided to use the more neutral term “perception of (oscillatory) self-motion” throughout this text.

2 Neglecting the fact that ISO2631-1:1997 only calculates the percentage of people who may vomit due to vertical motion, this percentage is given by ^1^/_3_*a*_w_√*T* with *a*_w_ the frequency weighted RMS acceleration, and *T* the exposure duration in seconds. For a sinusoidal displacement over 29 cm at a frequency of ~ 0.2 Hz, the RMS acceleration is 0.103 m/s^2^, the read frequency weighting *w*_f_ = 0.992 and *a*_w_ = 0.992 × 0.103 = 0.102 m/s^2^. This leads to a percentage of ^1^/_3_ × 0.102 ×√1800 = 1.4%.
