# Supplementary material for: Self-motion perception without sensory motion
Source: Exp Brain Res. 2022 Aug 20;240(10):2677–85. doi: 10.1007/s00221-022-06442-3 (PMC9510117; doi:10.1007/s00221-022-06442-3)
Supplement: Supplementary file 1 — Supplementary file1 (PDF 198 KB) [file 221_2022_6442_MOESM1_ESM.pdf]

Self-motion perception without sensory motion

A. J. C. Reuten[1,2]*, J. B. J. Smeets[1], M. H. Martens[3,4], J. E. Bos[2,1]
[1] Department of Human Movement Sciences, Vrije Universiteit Amsterdam, Amsterdam, The Netherlands
[2] Human Performance, The Netherlands Organization for Applied Scientific Research (TNO), Soesterberg, The Netherlands
[3] Traffic and Transport, The Netherlands Organization for Applied Scientific Research (TNO), The Hague, The Netherlands
[4] Department of Industrial Design, Eindhoven University of Technology, Eindhoven, The Netherlands
*a.j.c.reuten@vu.nl

ORCIDs

Reuten: 0000-0003-4641-4180
Smeets: 0000-0002-3794-0579
Martens: 0000-0002-1661-7019
Bos: 0000-0002-1494-6804

Supplementary Information

Temporal response traces of displacement and MISC class per participant

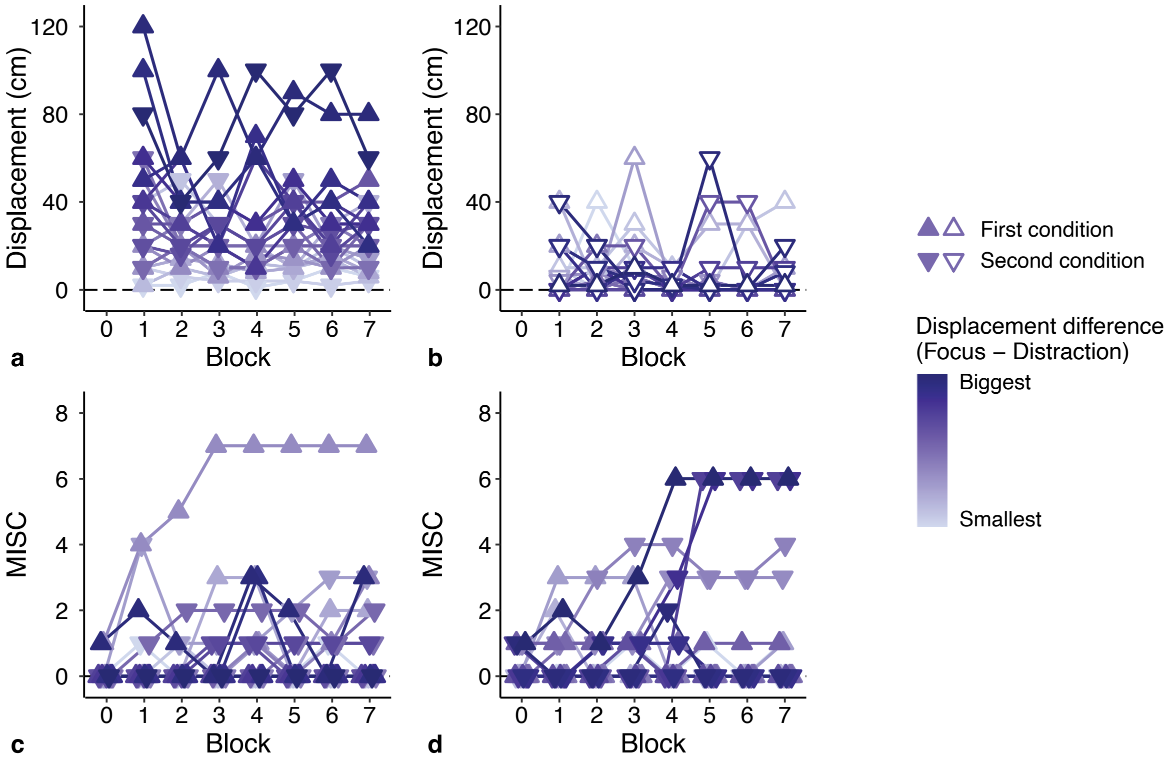

Fig. S1 The development of displacement (**a, b**) and MISC class (**c, d**) in the Focus and Distraction condition reported by individual participants. We used a gradient to contrast the participant with the biggest displacement difference between conditions (in dark purple) to the participant with the smallest displacement difference (in light purple). To visualize a possible effect of condition order, we used different symbols indicating the order of conditions for each participant

Exploratory correlational analyses

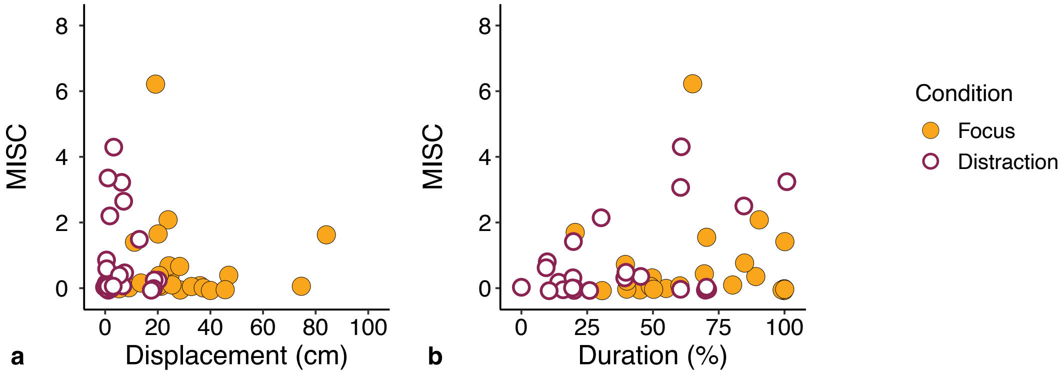

Fig. S2 We explored possible correlations between measures of self-motion perception and MISC class independent of condition. **(a)** There was no evidence for a correlation between the reported displacements and MISC classes (r_s = -0.04, p = 0.805), **(b)** neither between the reported motion durations (0% = never moved to 100% = always moved) and MISC classes (r_s = 0.12, p = 0.405). Jitter was added to help discriminate between individual data points
